# HIV prevalence in young people and children living on the streets, Kenya

**DOI:** 10.2471/BLT.18.210211

**Published:** 2018-11-06

**Authors:** Paula Braitstein, David Ayuku, Allison DeLong, Dominic Makori, Edwin Sang, Carren Tarus, Allan Kamanda, Pooja Shah, Edith Apondi, Juddy Wachira

**Affiliations:** a Dalla Lana School of Public Health, Division of Epidemiology, University of Toronto, 155 College Street, 5th floor, Toronto, Ontario M5T 3M7, Canada.; b School of Medicine, Moi University College of Health Sciences, Eldoret, Kenya.; c School of Public Health, Brown University, Providence, United States of America.; d Academic Model Providing Access to Healthcare (AMPATH), Eldoret, Kenya.; e Moi Teaching and Referral Hospital, Eldoret, Kenya.

## Abstract

**Objective:**

To obtain an estimate of the size of, and human immunodeficiency (HIV) prevalence among, young people and children living on the streets of Eldoret, Kenya.

**Methods:**

We counted young people and children using a point-in-time approach, ensuring we reached our target population by engaging relevant community leaders during the planning of the study. We acquired point-in-time count data over a period of 1 week between the hours of 08:00 and 23:00, from both a stationary site and by mobile teams. Participants provided demographic data and a fingerprint (to avoid double-counting) and were encouraged to speak with an HIV counsellor and undergo HIV testing. We used a logistic regression model to test for an association between age or sex and uptake of HIV testing and seropositivity.

**Findings:**

Of the 1419 eligible participants counted, 1049 (73.9%) were male with a median age of 18 years. Of the 1029 who spoke with a counsellor, 1004 individuals accepted HIV counselling and 947 agreed to undergo an HIV test. Combining those who were already aware of their HIV-positive status with those who were tested during our study resulted in an overall HIV seroprevalence of 4.1%. The seroprevalence was 2.7% (19/698) for males and 8.9% (23/259) for females. We observed an increase in seroprevalence with increasing age for both sexes, but of much greater magnitude for females.

**Conclusion:**

By counting young people and children living on the streets and offering them HIV counselling and testing, we could obtain population-based estimates of HIV prevalence.

## Résumé

**Objectif:**

Obtenir une estimation du nombre de jeunes et d'enfants qui vivent dans les rues d'Eldoret, au Kenya, ainsi qu'une estimation de la prévalence du virus de l'immunodéficience humaine (VIH) chez ces jeunes et ces enfants.

**Méthodes:**

Nous avons comptabilité ces jeunes et ces enfants à l'aide d'une approche ponctuelle, en veillant à toucher notre population cible grâce une coopération avec les responsables locaux concernés lors de la planification de l'étude. Nous avons obtenu des données de dénombrement ponctuel sur une période d'une semaine entre 08:00 et 23:00, grâce à un site fixe et à des équipes mobiles. Les participants ont fourni des données démographiques et leur empreinte digitale (pour éviter une double comptabilisation), et ont été encouragés à parler avec un conseiller en matière de VIH et à se soumettre à un dépistage du VIH. Nous avons utilisé un modèle de régression logistique pour déterminer l'existence d'un lien entre l'âge ou le sexe et le recours à un dépistage du VIH et la séropositivité.

**Résultats:**

Sur les 1419 participants admissibles comptabilisés, 1049 (73,9%) étaient des individus de sexe masculin dont l'âge médian était de 18 ans. Sur les 1029 individus ayant parlé avec un conseiller, 1004 ont accepté de recevoir des conseils sur le VIH et 947 de se soumettre à un dépistage du VIH. En combinant ceux qui étaient déjà au courant de leur séropositivité à ceux qui ont été soumis à un dépistage dans le cadre de notre étude, nous avons obtenu une séroprévalence générale du VIH de 4,1%. La séroprévalence était de 2,7% (19/698) chez les individus de sexe masculin et de 8,9% (23/259) chez les individus de sexe féminin. Nous avons constaté que la séroprévalence augmentait avec l'âge chez les deux sexes, mais que la magnitude était beaucoup plus importante chez les individus de sexe féminin.

**Conclusion:**

En comptabilisant les jeunes et les enfants qui vivent dans les rues et en leur proposant des conseils et un dépistage du VIH, nous avons pu obtenir des estimations démographiques de la prévalence du VIH.

## Resumen

**Objetivo:**

Obtener una estimación del número de jóvenes y niños que viven en las calles de Eldoret, Kenia, así como de la prevalencia de la inmunodeficiencia humana (VIH) entre ellos.

**Métodos:**

Se contaron a los jóvenes y los niños utilizando un enfoque puntual, asegurando que se llegase a la población objetivo mediante la participación de los líderes comunitarios correspondientes durante la planificación del estudio. Se adquirieron datos de conteo puntual en un periodo de 1 semana entre las 8:00 y las 23:00 horas, tanto de un sitio estacionario como de equipos móviles. Los participantes proporcionaron datos demográficos y una huella dactilar (para evitar el doble recuento) y se les animó a hablar con un asesor del VIH y a someterse a la prueba del VIH. Se utilizó un modelo de regresión logística para determinar la asociación entre la edad o el sexo y la aceptación de la prueba del VIH y la seropositividad.

**Resultados:**

De los 1419 participantes elegibles contados, 1049 (73,9 %) fueron hombres con una edad media de 18 años. De los 1029 que hablaron con un asesor, 1004 aceptaron recibir asesoramiento sobre el VIH y 947 aceptaron someterse a una prueba del VIH. La combinación de los que ya conocían su estado VIH-positivo y aquellos que se hicieron la prueba durante el estudio resultó en una seroprevalencia general del VIH del 4,1 %. La seroprevalencia fue del 2,7 % (19/698) para los hombres y del 8,9 % (23/259) para las mujeres. Observamos un aumento de la seroprevalencia con el aumento de la edad para ambos sexos, pero de mucha mayor magnitud para las mujeres.

**Conclusión:**

Al contar a los jóvenes y niños que viven en las calles y ofrecerles asesoramiento y pruebas del VIH, se puede obtener una estimación de la prevalencia del VIH basada en la población.

## ملخص

**الغرض:**

الحصول على تقدير لحجم وانتشار العوز المناعي البشري (HIV) بين الشباب والأطفال الذين يعيشون في شوارع إلدوريت، كينيا.

**الطريقة:**

قمنا ****باحتساب عدد الشباب والأطفال باستخدام نهج الوقت الزمني المحدد، مع ضمان وصولنا إلى السكان المستهدفين عن طريق إشراك قادة المجتمع المعنيين خلال التخطيط للدراسة. حصلنا على بيانات عدد لوقت زمني محدد عبر فترة أسبوع واحد بين الساعة 08:00 والساعة 23:00، من موقع ثابت وبواسطة فرق متنقلة. قدم المشاركون البيانات الديموغرافية والبصمة (لتجنب العد المزدوج) وتم تشجيعهم على التحدث مع مستشار العوز المناعي البشري والخضوع لاختبار للكشف عن هذا الفيروس. واستخدمنا نموذجاً للتحوف اللوجيستي لاختبار الارتباط بين العمر أو الجنس وتحمل اختبار العوز المناعي البشري والإيجابية للمصل.

**النتائج:**

من بين عدد 1419 مشاركاً مؤهلاً تم احتسابهم، كان 1049 (73.9٪) من الذكور وبلغ متوسط ​​أعمارهم 18 عاماً. من بين 1029 مشاركاً تحدثوا مع مستشار، قبل 1004 منهم المشورة بخصوص فيروس العوز المناعي البشري، ووافق 947 على الخضوع لاختبار فيروس العوز المناعي البشري. بالجمع بين هؤلاء الذين كانوا بالفعل على علم بحالة إصابتهم بفيروس العوز المناعي البشري، مع أولئك الذين تم اختبارهم خلال دراستنا، أسفر ذلك عن إيجابية مصلية إجمالية بنسبة 4.1٪. كانت الإيجابية المصلية 2.7٪ (19/698) للذكور، و8.9٪ (23/259) للإناث. قم بملاحظة زيادة في الإيجابية المصلية مع تزايد السن لكلا الجنسين، ولكن بدرجة أكبر بكثير بالنسبة للإناث.

**الاستنتاج:**

بإحصاء الشباب والأطفال الذين يعيشون في الشوارع، وتقديم المشورة والفحوص الخاصة بفيروس العوز المناعي البشرية، كان باستطاعتنا الحصول على تقديرات سكانية لانتشار العوز المناعي البشري.

## 摘要

**目的:**

旨在获取肯尼亚埃尔多雷特露宿街头的年轻人和儿童的人数及其人类免疫缺陷病毒 （即艾滋病毒，HIV） 患病率的估计值。

**方法:**

我们采用时点评级法对年轻人和儿童进行人数统计，确保在研究计划期间通过吸引相关社区领导者参与其中从而触及目标人群。我们从固定站点和移动团队获取了 1 周内每日 08:00 至 23:00 之间的时点统计数据。参与者提供了人口统计数据和指纹（以避免重复计算），同时被鼓励与艾滋病咨询师交谈并接受艾滋病毒检测。我们采用了逻辑回归模型来测试年龄或性别与艾滋病毒检测和血清阳性率之间的联系。

**结果:**

纳入计算的 1419 名符合条件的参与者中，1049 名（占 73.9％）是男性，中位年龄为 18 岁。在与咨询师交谈的 1029 人中，有 1004 人接受了艾滋病毒咨询，其中 947 人同意接受艾滋病毒检测。将已知艾滋病毒呈阳性的患者与在研究期间被检测出患有艾滋病毒的人数相加，得出总体艾滋病毒血清阳性率为 4.1％。男性血清阳性率为 2.7％（698 例中占 19 例），女性为 8.9％（259 例中占 23 例）。我们观察到血清阳性率随着两性年龄的增长而增加，但女性的涨幅更大。

**结论:**

通过统计街头年轻人和儿童人数并为他们提供艾滋病毒咨询和检测，我们可以获取基于人群的人类免疫缺陷病毒 (HIV) 患病率估计值。****

## Резюме

**Цель:**

Определение размера популяции детей и молодежи, которые живут на улицах Элдорета, Кения, и оценка уровня распространенности вируса иммунодефицита человека (ВИЧ) среди них.

**Методы:**

Авторы подсчитывали количество молодежи и детей по принципу «временной точки», а доступ к целевой популяции обеспечивался путем привлечения глав соответствующих общин на этапе планирования исследования. Данные учета по временным точкам собирались в течение 1 недели ежедневно с 8:00 до 23:00 как на стационарном участке наблюдения, так и мобильными группами. Участники опроса сообщали демографические данные и предоставляли отпечаток пальца во избежание двойного учета, и им рекомендовали пройти консультацию со специалистом по ВИЧ и ВИЧ-тестирование. Авторы применили модель логистической регрессии для проверки связи между возрастом и полом, с одной стороны, и охватом ВИЧ-тестированием и серопозитивностью, с другой.

**Результаты:**

Из 1419 участников исследования, учтенных авторами и отвечающих критериям, 1049 (73,9%) были мужского пола со средним возрастом 18 лет. Из 1029 человек, с которыми была проведена консультация, 1004 согласились на консультацию по ВИЧ и 947 согласились пройти тестирование на ВИЧ. С учетом тех, кто уже знал о своем ВИЧ-положительном статусе, и результатов тестирования, полученных в ходе исследования, общее количество лиц, сероположительных по ВИЧ, составило 4,1%. По данным тестирования, положительные результаты были выявлены у 2,7% юношей (19 из 698) и у 8,9% девушек (23 из 259). Чем старше были обследуемые, тем чаще встречался среди них сероположительный результат, но он всегда был выше для девушек.

**Вывод:**

Подсчет количества детей и молодежи, живущих на улицах, и предложение пройти консультацию и тестирование на ВИЧ позволили получить оценку заболеваемости ВИЧ в этой популяции.

## Introduction

The primary drivers of homelessness among children and young people globally are poverty, family conflict and child abuse and neglect.^1^ The United Nations International Children’s Emergency Fund (UNICEF) estimates that the number of children and young people living on the streets ranges from the tens to hundreds of millions.^2^^,^^3^ In an open letter to the United Nations and its Member States, over 175 community, philanthropic and other like-minded stakeholders issued a call that all children, including those out-of-school and/or without parental care, be represented in disaggregated data regionally, nationally and internationally.^4^^,^^5^ Primary prevention of homelessness among children and young people, and evidence-based policy and care for this population, requires surveillance to measure and monitor the problem through systematic data collection.^6^ There is a need to find empirically valid, generalizable and contextually relevant methods for counting young people living on the streets, especially in low- and middle-income countries.

Although no clear definition encompasses all the situations of young people living on the streets, understanding that their circumstances are fluid is critical. Definitions include: on the street (those on the street during the day, but returning to an adult caregiver or guardian at night); of the street (those on the streets both day and night); and children in homeless families, often the children of young homeless single mothers.^7^ In high-income settings, young people and children living on the streets are typically defined by their residential instability and precarious living arrangements, and may be referred to as homeless, runaways or unaccompanied youths.^8^ In this publication, we use the term street-connected youth to refer to those for whom the street is a central reference point, that is, one which plays a significant role in their everyday life.

Methods of counting homeless children in low- and middle-income countries have mainly involved headcount approaches.^9^^–^^12^ Headcounts involve selecting areas in which to perform counts with community stakeholders. However, numbers are acquired by simply counting the street children observed, sometimes for only one hour at a time, and subsequently agreeing with other stakeholders on the average or consensus number. This method is inaccurate however, because of double-, under- or overcounting as well as the subjectivity of those performing the count in deciding who appears to be homeless.

A more accurate method for counting homeless people, usually performed in high-income settings, is the point-in-time count.^13^^–^^16^ Such counts involve a census-type approach to identify and count homeless individuals over a given time period.

There are inherent difficulties in counting street-connected children and young people, including their mobility, invisibility and marginalization, which have resulted in wide-ranging estimates of the sizes of such populations and knowledge of them. The aim of this study was to adapt and pilot a point-in-time count of the street-connected youth population in the city of Eldoret, Kenya, incorporating opt-out human immunodeficiency virus (HIV) counselling and testing to estimate HIV prevalence within this population. We have reported on the risks for acquiring HIV and causes of death among the street-connected youth population in Kenya elsewhere, with HIV prevalence from convenience samples ranging from approximately 6% to 8%.^17^^–^^22^ These findings were however limited by non-differential selection biases in sampling and participation, limiting the validity and generalizability of results.

## Methods

### Study design and setting

We conducted our cross-sectional study during 4–10 September 2016 in the city of Eldoret, capital of the county of Uasin Gishu in Kenya.^23^ Approximately half of the population in Uasin Gishu live below the Kenyan poverty line^23^ of 1.90 United States dollars (US$) per day (at 2011 purchasing power parity).^24^ HIV prevalence among adults is 5%, consistent with the national average.^25^ As of 2011, Eldoret had a population of 300 000 and is the fifth-largest city in Kenya.^23^

We considered individuals to be eligible for participation if (i) they were younger than 30 years and (ii) they had spent more than 75% of their days and nights on the street or with other participants in a shared shelter for a minimum of the previous 3 months, to exclude those travelling or temporarily on the street. We did not randomly sample areas or people; instead, we ensured we reached our target population by enrolling peer community leaders during our preparation for the survey. The count was undertaken during term-time to exclude those youths who were only on the street during school holidays. We declined to count individuals who presented themselves but did not meet the inclusion criteria, however referred them to the relevant services, for example, the local sub-county hospital or various clinics in the referral hospital.

### Community mobilization and mapping

We engaged members of the community in the preparation of and planning for the count nearly 3 months before its execution. The first stage included discussions with known community leaders, including directors and other key staff of relevant community-based organizations, and other friends and advocates of the street-connected youth population (e.g. some local business leaders). We visited locations important to the street-connected youth under the guidance of existing peer navigators,^26^ a specialized social worker, and both male and female street-connected youth community leaders. The locations around Eldoret at which participants congregate and shelter include peri-urban settlements, waste disposal sites and other locations either identified by the community leaders or known to the research team (e.g. an empty lot used as a gathering place). *Mabaraza* (traditional community assemblies) were held with the various gatherings of participants around the city.^27^ Information was provided about why the count was important, what it would require of each participant and the services being offered, including a single serving of bread and milk (worth approximately US$ 1) to people being counted.

Personnel were recruited from community and facility sources. The HIV counsellors, clinical staff and data assistants were seconded from the local HIV programme or other research projects to provide services for the duration of the count. Senior project staff, clinical supervisors and key community members led training of all personnel at the referral hospital, following numerous planning meetings. Training consisted of ethics certification, study purpose and design, standard operating procedures and work flow for the various teams. Other preparations included engaging community leaders, mapping specific counting locations, and specifying dates and times for counting. Community mobilization included discussions with the County Children’s Officer and police and security officials to minimize the potential for stigmatization and police violence.^28^

### Aspects of the count

We conducted our point-in-time count over a period of seven consecutive days during 08:00 to 23:00 each day. We maintained both a stationary counting site (that moved once after the first site was considered saturated) and a mobile team who visited relevant locations at the appointed days and times. Saturation of a site was determined when no new individuals had been counted for at least one day at the stationary site or one hour at the mobile sites.

We used four teams, each including 10–15 people, comprised of two team leaders (an established community leader and another from the research team), enumerators, data assistants, HIV counsellors certified by the health ministry (who also performed HIV testing), clinicians (who provided onsite first aid and referrals to health care and other available services), volunteers who distributed bread and milk to each individual counted, and other volunteer assistants who ensured individuals proceeded smoothly through the process. Everyone counted was issued with a laminated hospital card with their handwritten unique identifier, name, sex and date of birth, which could be used as a proxy form of identification (which most lack) to gain access to the hospital when in need or if referred during the study.

We made several adaptations to the original point-in-time count^29^ by holding the count over one week and over both daytime and evening hours until 23:00, having both stationary and mobile counting sites, and integrating HIV counselling and testing within the count. Instead of counting individuals in homeless shelters (which do not exist in Eldoret), we included individuals on the street during the day, but off the street with family or friends at night.

### The count procedure

When individuals first presented themselves, we recorded each participant’s digital fingerprint using a ZK-US-10 Biometric fingerprint reader (ZKTeco USA LCC, Fairfield, United States of America) to prevent double-counting. We only stored fingerprint data in the fingerprint reader and without a personal identifier. We used the sequential number assigned by the reader equipment to each fingerprint as a study identifier for the individual. If individuals presented themselves for a second time, the machine issued a warning about the fingerprint already being read. We then interviewed participants, obtaining data including name (whether legal, made up or a nickname), date of birth (confirmed or estimated, being 01/01/year if estimated), sex and where they slept at night (home with parent or guardian, home with a sibling or other relative, in a shared shelter with other street youth, on the street, in barracks or other). We recorded all count data using Excel (Microsoft, Redmond, USA). 

Participants were introduced to the peer navigators, whose job was to increase the uptake of both HIV testing and of care and treatment if positive, and the uptake of other health services as needed (e.g. psychological counselling).^26^ To protect the privacy and confidentiality of participants, we encouraged them to speak with the peer navigators if they had received an HIV-positive diagnosis, either previously or through our survey, and wanted help to access care. At this stage, we also offered the participants the opportunity to speak with an HIV counsellor. 

### HIV counselling and testing

Of those individuals who agreed to speak with an HIV counsellor, we recorded whether or not they had ever been previously tested, their HIV status at the last test and, if diagnosed HIV positive, whether they were receiving care. Individuals who were previously HIV negative or with a HIV status unknown, were invited to receive HIV counselling and testing during the interaction, which took place either in a counselling tent at the stationary sites or at a location where the HIV counsellors had set themselves up some distance away for mobile sites. Participants had the possibility to refuse counselling but accept HIV testing. We conducted HIV testing according to the national algorithm,^30^ first using a Colloidal Gold Diagnostic Kit (KHB, Shanghai, China) to detect the presence of the HIV (1+2) antibody (requiring 40 µL of plasma or whole blood), and confirming positive results using a First Response HIV Card Test (Premier Medical Corporation, Mumbai, India). In the case of contradictory results, we determined HIV status using a Uni-Gold reagent assay (Trinity Biotech, Bray, Ireland).

Results were documented by the HIV counsellors for their routine reporting systems (including identification) separately from results for this research (excluding identification). 

### Statistical methods

We defined HIV prevalence as the total of individuals known to be positive before the survey or newly diagnosed while participating in the study. We used a logistic regression model to measure the relationship between age category or sex and HIV testing uptake or seropositivity. We used a generalized additive model to confirm a nonlinear relationship between the log-odds of agreeing to HIV testing or of seropositivity and age, and to determine whether there were separate age thresholds for females and males at which increases in HIV prevalence were observed. All analyses were undertaken using R software (R Foundation, Vienna, Austria).^31^

### Ethics

Our study was reviewed and approved by the Moi University/Moi Teaching and Referral Hospital Institutional Research Ethics Committee and the University of Toronto’s Research Ethics Board. We obtained a waiver of guardian consent for minors, and a waiver of written consent and assent for participants to be counted and offered HIV counselling and testing. Verbal consent and assent were obtained for the count and HIV counselling and testing separately. Infants who were brought to the count by a street-connected parent were both counted and tested for HIV with the verbal consent of their parent. Personal identifying information from the count was not linked to the HIV testing uptake or results. The study was of minimal risk and had potential benefits to participants, including onsite first aid, referrals, psychological counselling and HIV counselling and testing. The community agreed that unwilling participants would simply not attend. Individuals could receive HIV counselling and testing, first aid and psychological support without participating in the count. Equally, individuals could participate in the count without being required to receive HIV counselling or testing.

## Results

**Fig. 1:**
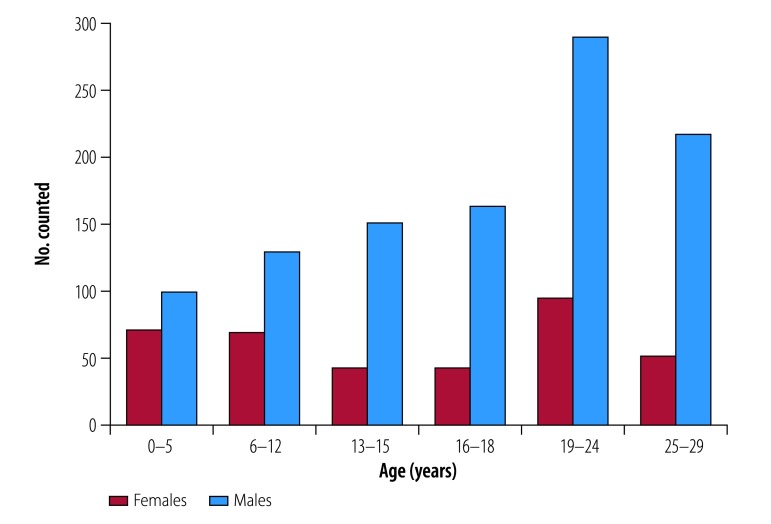
Age and sex distribution of counted young people and children living on the streets, Eldoret, Kenya, September 2016

We counted a total of 1903 individuals; 484 (25.4%) were older than 29 years and excluded from further analysis. Of the 1419 participants included in the study, 1049 (73.9%) were male with a median age of 18 years (Table 1). There were 170 children aged 5 years or younger, 198 children aged 6–12 years, 398 adolescents aged 13–18 years and 384 young people aged 19–24 years (Fig. 1). Overall, 368 (25.9%) of the population counted were younger than 13 years. Although our target was street-connected youth, we also counted 269 street-connected adults aged 25–29 years. We counted a total of 370 girls and women, 224 (60.5%) of whom were 18 years or younger. Although only 1027 (72.4%) individuals agreed to speak with an HIV counsellor, 1029 made it to the counselling tent due to attendance of the two participants whose agreement was not recorded; 1004 out of 1029 (97.6%) also agreed to HIV counselling. A similar proportion of total male participants (734/1049, 70.0%) accepted HIV counselling as females (270/370, 73.0%) but, due to the higher proportion of male participants, our HIV testing cohort was 26.1% (247/947) female and 73.9% (700/947) male. There were no significant differences in the numbers of those who accepted HIV counselling and testing in terms of age (Table 1) or usual place of sleeping. Of the 1027 who agreed to speak with a counsellor, 211 (20.5%) slept at home with their parents or a guardian, 98 (9.5%) with a sibling or other relative, 149 (14.5%) in a rental with friends, 207 (20.2%) on the streets, 117 (11.4%) in barracks and 245 (23.9%) at some other location. 

**Table 1:** Sociodemographic characteristics of the sampled young people and children living on the streets in Eldoret, Kenya, September 2016

Covariate	No. (%)			
	Total (*n* = 1419)^a^	Children aged < 15 years (*n* = 497)	Youths aged 15–24 years (*n* = 653)	Adults aged 25–29 years (*n* = 269)
**Sex**				
Male	1049 (73.9)	321 (64.6)	511 (78.3)	217 (80.7)
Female	370 (26.1)	176 (35.4)	142 (21.7)	52 (19.3)
**Regular sleeping location**				
Home with parents or legal guardian	284 (20.0)	237 (47.7)	39 (6.0)	8 (3.0)
Home with sibling or other relative	142 (10.0)	33 (6.6)	64 (9.8)	45 (16.7)
Rental with friends	210 (14.8)	33 (6.6)	139 (21.3)	38 (14.1)
Streets (sidewalks, market, veranda)	291 (20.5)	79 (15.9)	155 (23.7)	57 (21.2)
Barracks	160 (11.3)	33 (6.6)	92 (14.1)	35 (13.0)
Other (specified)	332 (23.4)	82 (16.5)	164 (25.1)	86 (32.0)
**Agreed to talk with HIV counsellor **				
Yes	1027 (72.4)	372 (74.8)	465 (71.2)	190 (70.6)
No	390 (27.5)	123 (24.7)	188 (28.8)	79 (29.4)
Missing	2 (0.1)	2 (0.4)	0 (0.0)	0 (0.0)

HIV: human immunodeficiency virus.

^a^ Median age (interquartile range) of total, children, youths and adults: 18 (12–23), 9 (3–13), 19 (17–22) and 27 (26–28) years.

Note: Inconsistencies arise in some values due to rounding.

Table 2 summarizes the HIV counselling, testing and seroprevalence characteristics of the population by age and sex. Briefly, 19/1029 (1.8%) of the population who agreed to speak with a counsellor already knew they were HIV-positive; of those, 16 (84.2%) self-reported that they were already receiving HIV care. Of the 1004 individuals who agreed to HIV counselling, 985 (98.1%) had previously tested HIV-negative or were unaware of their HIV status. Of the 947 HIV tests conducted, which included 10 participants who had refused HIV counselling, 23 (2.4%) tested HIV positive.

**Table 2:** HIV testing and counselling outcomes of young people and children living on the streets in Eldoret, Kenya, September 2016

Covariate	No. (%)							
	Total		Children aged < 15 years		Youths aged 15–24 years		Adults aged 25–29 years	
	Males (*n* = 749)	Females (*n* = 280)	Males (*n* = 242)	Females (*n* = 131)	Males (*n* = 377)	Females (*n* = 106)	Males (*n* = 130)	Females (*n* = 43)
**Accepted HIV counselling**								
Yes	734 (98.0)	270 (96.4)	230 (95.0)	122 (93.1)	374 (99.2)	105 (99.1)	130 (100.0)	43 (100.0)
No	15 (2.0)	10 (3.6)	12 (5.0)	9 (6.9)	3 (0.8)	1 (0.9)	0 (0.0)	0 (0.0)
Missing	0 (0.0)	0 (0.0)	0 (0.0)	0 (0.0)	0 (0.0)	0 (0.0)	0 (0.0)	0 (0.0)
**HIV status**								
Known positive	5 (0.7)	14 (5.0)	0 (0.0)	0 (0.0)	2 (0.5)	5 (4.7)	3 (2.3)	9 (20.9)
Not known or negative^a^	743 (99.2)	265 (94.6)	242 (100.0)	131 (100.0)	375 (99.5)	100 (94.3)	126 (96.9)	34 (79.1)
Missing	1 (0.1)	1 (0.4)	0 (0.0)	0 (0.0)	0 (0.0)	1 (0.9)	1 (0.8)	0 (0.0)
**Of those known positive, receiving care**								
Yes	3 (60.0)	13 (92.9)	NA	NA	1 (50.0)	5 (100.0)	2 (66.7)	8 (88.9)
No	2 (40.0)	1 (7.1)	NA	NA	1 (50.0)	0 (0.0)	1 (33.3)	1 (11.1)
Missing	0 (0.0)	0 (0.0)	0 (0.0)	0 (0.0)	0 (0.0)	0 (0.0)	0 (0.0)	0 (0.0)
**Of those previously diagnosed negative or unaware of HIV status, accepted testing^b^**								
Yes	700 (94.1)	247 (92.9)	215 (88.8)	116 (88.5)	363 (96.8)	99 (98.0)	122 (96.1)	32 (94.1)
No	44 (5.9)	19 (7.1)	27 (11.2)	15 (11.5)	12 (3.2)	2 (2.0)	5 (4.0)	2 (5.9)
Missing	0 (0.0)	0 (0.0)	0 (0.0)	0 (0.0)	0 (0.0)	0 (0.0)	0 (0.0)	0 (0.0)
**Of those tested, HIV test results**								
Positive	14 (2.0)	9 (3.6)	2 (0.9)	1 (0.9)	8 (2.2)	6 (6.1)	4 (3.3)	2 (6.3)
Negative	679 (97.0)	236 (95.5)	211 (98.1)	115 (99.1)	350 (96.4)	91 (91.9)	118 (96.7)	30 (93.8)
Indeterminate	3 (0.4)	0 (0.0)	0 (0.0)	0 (0.0)	3 (0.8)	0 (0.0)	0 (0.0)	0 (0.0)
Missing	4 (0.6)	2 (0.8)	2 (0.9)	0 (0.0)	2 (0.6)	2 (2.0)	0 (0.0)	0 (0.0)
**HIV prevalence^c^**	19 (2.7)	23 (8.9)	2 (0.9)	1 (0.9)	10 (2.8)	11 (10.8)	7 (5.6)	11 (26.8)

HIV: human immunodeficiency virus; NA: not applicable.

^a^ These numbers included those known to be HIV negative, those who had never been tested and those who may have tested negative many months previously and felt they had recently been exposed.

^b^ These numbers include 10 participants who had declined HIV counselling.

^c^ Including previously known and newly tested positive individuals, excluding indeterminate results and missing data.

Note: Inconsistencies arise in some values due to rounding.

**Fig. 2:**
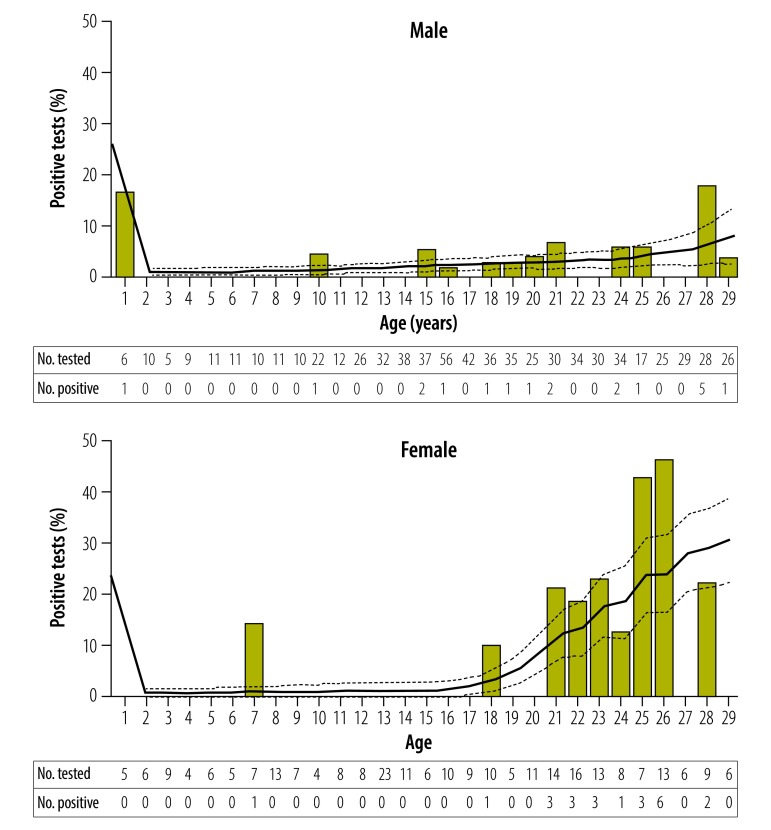
HIV seroprevalence of young people and children living on the streets, Eldoret, Kenya, September 2016

HIV: human immunodeficiency virus; SE: standard error.

Notes: The bars represent raw data and the solid curve is a prediction of prevalence from the generalized additive model. Dashed curves represent a SE of 1*σ*.

Combining those who were already aware of their HIV-positive status with those newly diagnosed resulted in an HIV seroprevalence of 4.4% (42/957) among those who agreed to speak with a counsellor. When we considered sex separately, the seroprevalence for males was 2.7% (19/698) and for females was 8.9% (23/259). HIV seroprevalence in males increases from 2.8% in the age group 15–24 years to 5.6% in the age group 25–29 years; HIV seroprevalence in females increases more between these age categories from 10.8% to 26.8% (Table 2). We did not observe any significant difference in HIV testing uptake between males and females after adjusting for age (Table 3), or any significant difference in HIV seroprevalence between males and females in the age category of younger than 15 years. However, women aged 15–24 years and 25–29 years were above four and six times more likely to be HIV positive than men, respectively (odds ratio, OR: 4.23; 95% confidence interval, CI: 1.74–10.27 and OR: 6.18; 95% CI: 2.21–17.29; Table 3). Fig. 2 illustrates a stable but increasing HIV seroprevalence among males with increasing age, however depicts an escalation of greater magnitude among females with increasing age.

**Table 3:** Difference in uptake of HIV testing and HIV seroprevalence of young people and children living on the streets, Eldoret, Kenya, September 2016

Covariate	OR (95% CI)	
	Agreed to HIV test^a^	HIV seroprevalence^b^
**Sex**		
Male	1.00	1.00
Female	1.02 (0.58–1.80)	4.38 (2.30–8.34)
**Age**		
< 15 years	1.00	1.00
15–24 years	4.20 (2.24–7.85)	6.65 (1.95–22.74)
25–29 years	2.80 (1.22–6.39)	16.81 (4.80–58.8 7)
**Sex and age**		
< 15 years		
Male	NA	1.00
Female	NA	0.92 (0.08–10.23)
15–24 years		
Male	NA	1.00
Female	NA	4.23 (1.74–10.27)
25–29 years		
Male	NA	1.00
Female	NA	6.18 (2.21–17.29)

CI: confidence interval; HIV: human immunodeficiency virus; NA: not applicable; OR: odds ratio.

^a^ Excluding those already known to be HIV-positive.

^b^ A combination of those already known to be HIV positive plus those who tested positive during this study, excluding indeterminate results and missing data.

Note: For the uptake of HIV testing, sex and age categories are adjustment variables.

## Discussion

Our data provide an estimate of the magnitude of the street-connected youth population and highlight especially vulnerable groups within them who should be prioritized for care and protection, notably those younger than 15 years and all females. Our population-level estimate of HIV seroprevalence in young people and children living in the streets of Eldoret is consistent with the average seroprevalence of 4.7% in the general adult population of the county.^32^ We also found that street-connected females were much more likely to be HIV positive than males, consistent with the generalized epidemic among adults in sub-Saharan Africa.^25^ Compared with a county-wide prevalence of 6.7% in females and 4.0% of males,^32^ we observed a greater difference between female and male street-connected people.^33^

A description of a headcount undertaken over a period of 10 days in Mwanza, United Republic of Tanzania, reported a higher number of young people living on the streets (1888 versus 1419), but a smaller number of females (305 versus 370).^11^ With a population of over 400 000, Mwanza is United Republic of Tanzania’s second largest city.^34^ We may have reached a broader segment and more reliable (in terms of reproducibility) number of the target population compared with an observational headcount or a single-night point-in-time count. Street-connected youth populations are mobile and follow market schedules, community programmes and other opportunities to make money or otherwise meet basic needs, so having both stationary and mobile counting stations is crucial. Females are likely to be in different places and at different times from males; for example, while the males may be working (e.g. using luggage carts for money around the bus stations or finding parking spaces for drivers), females are more likely to be found begging in the downtown centre. There may be tensions between different groups of street-connected youth because of interpersonal conflicts or competition over resources, preventing some groups of youths from being counted at the same time as others. We therefore developed targeted and tailored strategies to reach as many as possible.

Our study had several strengths. Instead of randomly sampling a population or area, we carefully planned our study to target the most relevant street-connected population through our strong relationships with them and with the County Children’s Officer, health-care providers, relevant community organizations and the police. We avoided the possibility of overcounting by using electronic fingerprinting. As part of our strategy to safeguard the rights of the city’s street-connected youth population^35^ we offered various services, even to those who refused to be counted. Finally, we conducted the count over a 7-day period with strategically placed stationary and mobile counting points, during both daylight and evening hours until 23:00.

Our study also had limitations. This was a cross-sectional study, making it impossible to investigate any causal relationship between being street-connected and HIV seroprevalence. Due to the stigma attached to being homeless, some children did not want to self-identify as being street-connected;^18^ our count may therefore have missed some individuals who did not want to come forward. We did not assess important issues such as substance abuse or HIV risk factors, which participants may have been uncomfortable discussing, as our focus was counting the population and determining HIV prevalence. Participants’ self-reported age may have been inaccurate, as many do not know their exact birth date. Finally, as this was conducted in a single city, the findings may not be generalizable to other cities in the region which may differ in background HIV prevalence and other relevant characteristics such as population.

In conclusion, we have demonstrated that it is possible to obtain population-based estimates of HIV prevalence in a low-income setting, using generalizable and reproducible methods in terms of community engagement and data collection, by counting street-connected youth population and offering HIV counselling and testing. Such basic surveillance activities are vital to monitor and address child and youth homelessness. This population requires public health authorities, community and health-service providers and policy-makers to implement programmes to prevent premature mortality among them and to uphold their rights.^22^
